# *synaptojanin1* Is Required for Temporal Fidelity of Synaptic Transmission in Hair Cells

**DOI:** 10.1371/journal.pgen.1000480

**Published:** 2009-05-08

**Authors:** Josef G. Trapani, Nikolaus Obholzer, Weike Mo, Susan E. Brockerhoff, Teresa Nicolson

**Affiliations:** 1 Howard Hughes Medical Institute, Oregon Health and Science University, Portland, Oregon, United States of America; 2 Oregon Hearing Research Center and Vollum Institute, Oregon Health and Science University, Portland, Oregon, United States of America; 3 Department of Biochemistry, University of Washington School of Medicine, Seattle, Washington, United States of America; University of Göttingen, Germany

## Abstract

To faithfully encode mechanosensory information, auditory/vestibular hair cells utilize graded synaptic vesicle (SV) release at specialized ribbon synapses. The molecular basis of SV release and consequent recycling of membrane in hair cells has not been fully explored. Here, we report that *comet*, a gene identified in an ENU mutagenesis screen for zebrafish larvae with vestibular defects, encodes the lipid phosphatase Synaptojanin 1 (Synj1). Examination of mutant *synj1* hair cells revealed basal blebbing near ribbons that was dependent on Cav1.3 calcium channel activity but not mechanotransduction. Synaptojanin has been previously implicated in SV recycling; therefore, we tested synaptic transmission at hair-cell synapses. Recordings of post-synaptic activity in *synj1* mutants showed relatively normal spike rates when hair cells were mechanically stimulated for a short period of time at 20 Hz. In contrast, a sharp decline in the rate of firing occurred during prolonged stimulation at 20 Hz or stimulation at a higher frequency of 60 Hz. The decline in spike rate suggested that fewer vesicles were available for release. Consistent with this result, we observed that stimulated mutant hair cells had decreased numbers of tethered and reserve-pool vesicles in comparison to wild-type hair cells. Furthermore, stimulation at 60 Hz impaired phase locking of the postsynaptic activity to the mechanical stimulus. Following prolonged stimulation at 60 Hz, we also found that mutant *synj1* hair cells displayed a striking delay in the recovery of spontaneous activity. Collectively, the data suggest that Synj1 is critical for retrieval of membrane in order to maintain the quantity, timing of fusion, and spontaneous release properties of SVs at hair-cell ribbon synapses.

## Author Summary

Ribbon synapses are found in the ear and eye and facilitate the transmission of sensory information to the brain. In hair cells of the ear, the molecules required for ribbon function have not been fully explored. Zebrafish are ideal for investigating molecular components of these specialized synapses because of the ability to study ribbon function using genetic, cellular, and physiological methods. Here, we explore the role of the lipid phosphatase Synaptojanin at the hair cell synapse. Synaptojanin has been previously implicated in synaptic vesicle recycling in conventional synapses, and we also find that the number of synaptic vesicles are reduced in mutant *synaptojanin* hair cells. Mutant *synaptojanin* larvae have obvious equilibrium defects, and our electrophysiological recordings revealed that synaptic transmission from hair cells to neurons projecting to the brain is impaired in terms of both rate and accuracy. When stimulated at high frequency or for prolonged periods, mutant *synaptojanin* hair cells release vesicles out of phase with mechanical stimuli, thus compromising the transfer of sensory information to the brain.

## Introduction

Vertebrate hair cells generate graded receptor potentials in response to mechanical stimuli, resulting in neurotransmitter release with high temporal accuracy and fidelity. Precise timing of release supports the phase locking of post-synaptic activity to stimuli in the kilohertz range [1]. These high rates of transmitter release require the specialized ribbon synapse [2]–[4]. For example, in hair cells of the mouse inner ear, ribbon synapses support remarkable rates of exocytosis approaching 1000 SV/sec [5],[6]. Such high rates are probably achieved by ribbon-mediated rapid exocytosis of a large pool of readily releasable vesicles [3], [7]–[10].

The role of the ribbon in orchestrating timed SV release is not fully characterized. Perhaps specialized, ribbon-associated proteins are required for accurate timing of release. Additionally, the availability of vesicles at hair-cell ribbons may be necessary and sufficient for both release rate and timing. A recent modeling study of ribbon transmission depicts how increased numbers of docked, releasable vesicles reduces both the latency of release and the associated temporal jitter at ribbon synapses [11]. To maintain a steady supply of SVs at the ribbon, hair cells maintain a large reserve pool of vesicles. Thus, thousands of SVs can be released in response to strong stimulation with membrane turnover approaching a quantity of half the cell surface area within seconds [12],[13]. It has been shown that disruption in vesicle recycling results in a reduced transmission at neuromuscular junctions [14]. An effect on temporal precision of release in hair-cell synapses has not been investigated in vivo.

A key player in vesicle recycling is synaptojanin1 (Synj1; reviewed by [15]). Synj1 is a multidomain lipid-phosphatase that contains a sac1-like phosphatase domain, a 5-phosphatase domain, and a proline-rich domain (PRD). Synj1 regulates phosphoinositide turnover, especially that of phosphatidylinositol (4,5) bisphosphate (PI(4,5)P2), resulting in the shedding of the clathrin coat on internalized vesicles [16],[17]. In zebrafish, *synj1*-deficient *nrc* (no optokinetic response ‘c’) mutants have visual defects and floating synaptic ribbons in photoreceptors, but not bipolar cells [18],[19]. Van Epps et al. noted an affect on balance, but did not examine the expression and role of *synj1* in hair cells [19].

We report here that the *comet* gene, which was isolated during a large-scale mutagenesis screen, encodes *synj1*. However, mutant zebrafish larvae display balance defects and a corresponding impaired vestibulo-ocular reflex. Given the previously described role of *synj1* in SV recycling, we examined synaptic transmission and vesicle pools in mutant hair cells. We found a decrease in spike rate along with a decrease in number of ribbon-associated SVs. Our recordings also revealed an impairment of phase locking in *synj1* mutants and a striking delay in the return of spontaneous release of SVs following periods of prolonged stimulation in mutants. Taken together, these results suggest that active maintenance of vesicle pools is required for robust and accurate timing of release of SVs in hair cells.

## Results

### Genetic Lesions and Expression of *synj1* in Hair Cells

**Figure 1:**
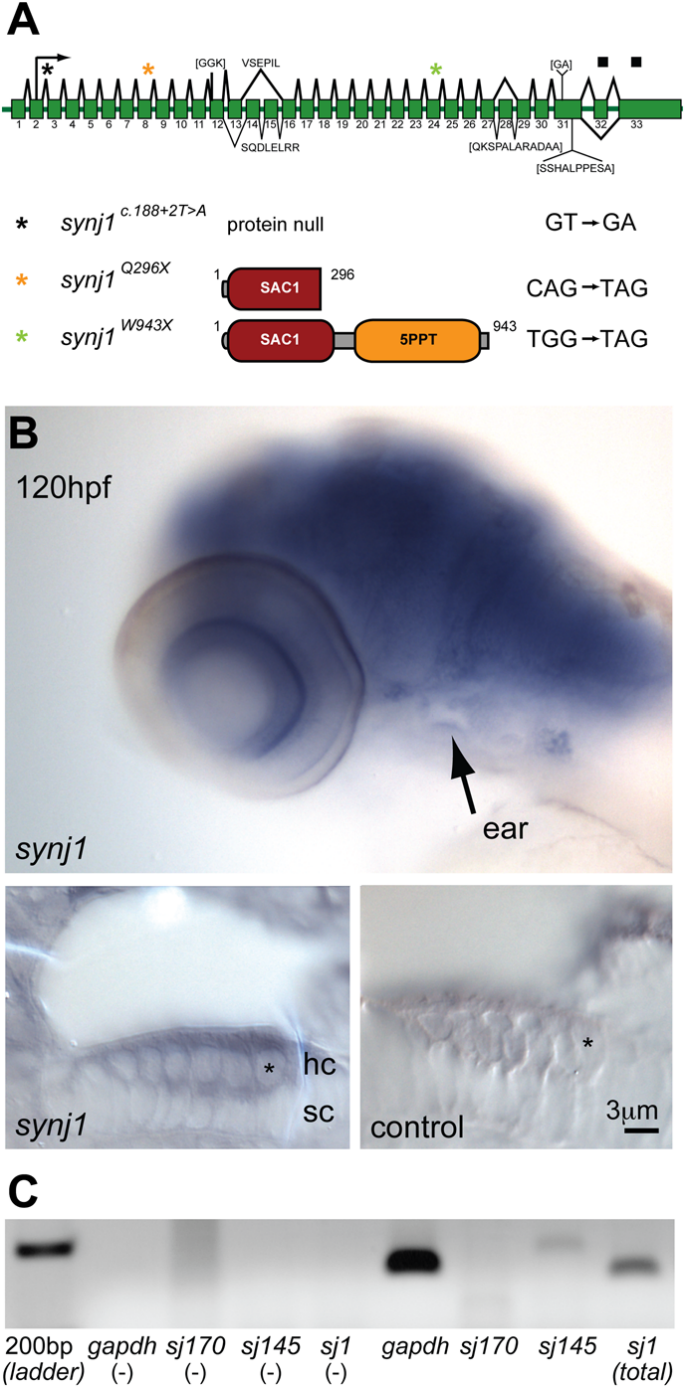
*synj1* is mutated in *comet* mutants. A) Splice diagram of the zebrafish *synj1* gene indicating alternate splicing of exons 13, 14 and 15, which can lead to the inclusion or exclusion of other indicated sequences (brackets). The diagram was assembled from collective *synj1* sequencing data. Three new mutations of *synj1* include deletion of the translation start site (donor splice site inactivation), and nonsense mutations, resulting in truncations at amino acid 296, or amino acid 943. A color-coded asterisk above the splice diagram indicates the position of the respective mutation in the transcript, and the expected protein product is depicted after the allele identifier, followed by the mutated nucleotide sequence. B,C) Expression of *synj1* in inner ear and neuromast hair cells. B) *synj1* expression pattern in the developing larva as shown by *in situ* hybridization. At 120 hpf, *synj1* is expressed at high levels in the brain, eye and inner ear (arrow; upper panel). Hair-cell specific expression (asterisk) was observed in transverse thick sections of the ear at 100× magnification; both anti-sense *synj1* and sense control label are shown (left and right lower panels, respectively). C) RT-PCR for *synj1* from isolated neuromast hair-cells amplified the short (*synj1*-145) isoform. The panel depicts, from left to right, four control reactions (no template) and four hair-cell mRNA template reactions. *gapdh* mRNA was used as a positive control (band at 151 bp). Specific primer pairs were used to selectively amplify the long and short isoforms *sj170* (101 bp expected band size) and *sj145* (200 bp), respectively. The *sj1* indicates total *synj1* mRNA (140 bp).

Three *comet* mutants were isolated in a large-scale chemical mutagenesis screen for larvae with balance defects. To characterize the mutant *comet* phenotype at the molecular level, we mapped the *comet* gene and noted that the recessive lesions mapped closely to a previously identified locus of the *nrc* gene [19]. The *nrc* mutant has a nonsense mutation in *synj1*, leading to visual defects. We confirmed the identity of *comet* as *synj1* by complementation analysis with *nrc* heterozygous fish. Sequencing revealed that two alleles, *synj1^Q296X^* and *synj1^W943X^*, contain nonsense mutations that lead to truncations of the protein product within the first and after the second phosphatase domain, respectively (Figure 1A). The genomic lesion of the third allele, *synj1* ^c.188+2T>A^, inactivates the donor splice site of exon 2 that contains the translation start site (ATG), thereby leading to the deletion of exon 2 and presumably no protein product (Figure 1A). The *synj1^Q296X^* allele causes a truncation within the SAC1 domain similar to the original *nrc* allele (R499X), which results in undetectable levels of Synj1 protein in larval brain extracts [19]. Because differences in behavior, cellular or physiological phenotypes could not be detected among larvae carrying the three alleles, we conducted our experiments with *synj1^Q296X^* mutants.

Next we investigated *synj1* expression in the larval auditory/vestibular system. In 96–120 hours post fertilization (hpf) zebrafish larvae, we detected *synj1* mRNA expression in the central nervous system: foremost in the brain, retina, and in the ear (Figure 1B, upper panel). Sections of the developing ear showed expression of *synj1* mRNA in hair cells (Figure 1B, lower left panel). In addition to visualizing *synj1* expression by *in situ* hybridization, we injected a plasmid with a 6.5 kb fragment of the *synj1* promoter driving EGFP and observed transient EGFP expression in the nervous system and in hair cells of both the ear and lateral line (data not shown). Our results confirm a previous report of expression of *synj1* in zebrafish hair cells using microarray analysis [20]. Using RT-PCR on tissue isolated from neuromasts, we detected the *synj1*-145 but not the *synj1*-170 isoform, suggesting that the short splice variant is specific to hair cells (Figure 1C). As positive controls, PCR reactions using random decamer mRNA from whole larvae showed robust amplification with each primer set (data not shown). In addition to *in situ* hybridization and RT-PCR of isolated neuromasts, we attempted to label Synj1 with antibodies, but were not successful with either published [19] or newly generated antibodies.

**Figure 2:**
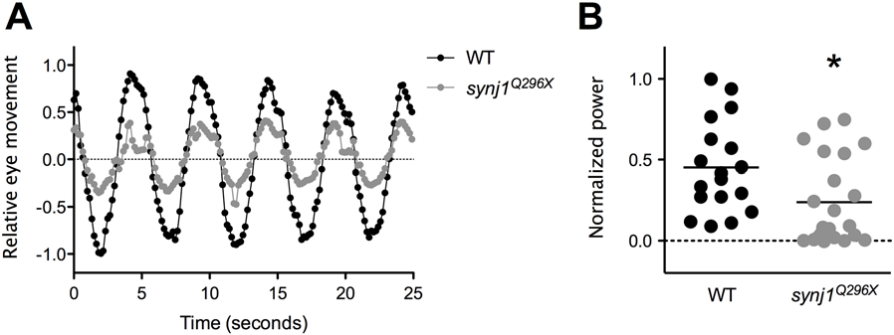
The vestibulo-ocular reflex is reduced in *synj1* mutants, indicating a balance defect. Shown in (A) are average relative eye movements of both eyes in wild type (WT; black circles) and *synj1^Q296X^* mutant (grey circles) larvae in response to 0.2 Hz stimulation (n>9). B) Plot of power at 0.2 Hz for both eyes for each individual WT (black circles) and mutant (grey circles) larva shown in (A). All values were normalized to the largest WT value. The mean power is represented by the black horizontal lines.

The characteristic tilting posture displayed by *synj1* mutant larvae is indicative of vestibular dysfunction. When mutant larvae were continuously swirled in a Petri dish of water they adopted more severely affected postures, including floating upside down. To determine the extent to which vestibular function was affected in *synj1^Q296X^* mutants, we tested the vestibulo-ocular reflex (VOR) in 120 hpf larvae. The VOR is an involuntary compensatory eye movement in response to stimulation of the vestibular system. The VOR in response to rotation of the head was significantly reduced in *synj1^Q296X^* mutants (Figure 2A). We observed a 48% reduction of mean relative eye movement (quantified by the total power at 0.2 Hz) in mutants compared to wild-type siblings (Figure 2B). Although the response is clearly present, these data suggest that transmission of vestibular information in *synj1* mutants is impaired.

### Basal Blebbing in Mutant *synj1* Hair Cells Is Dependent on Cav1.3 Channels

**Figure 3:**
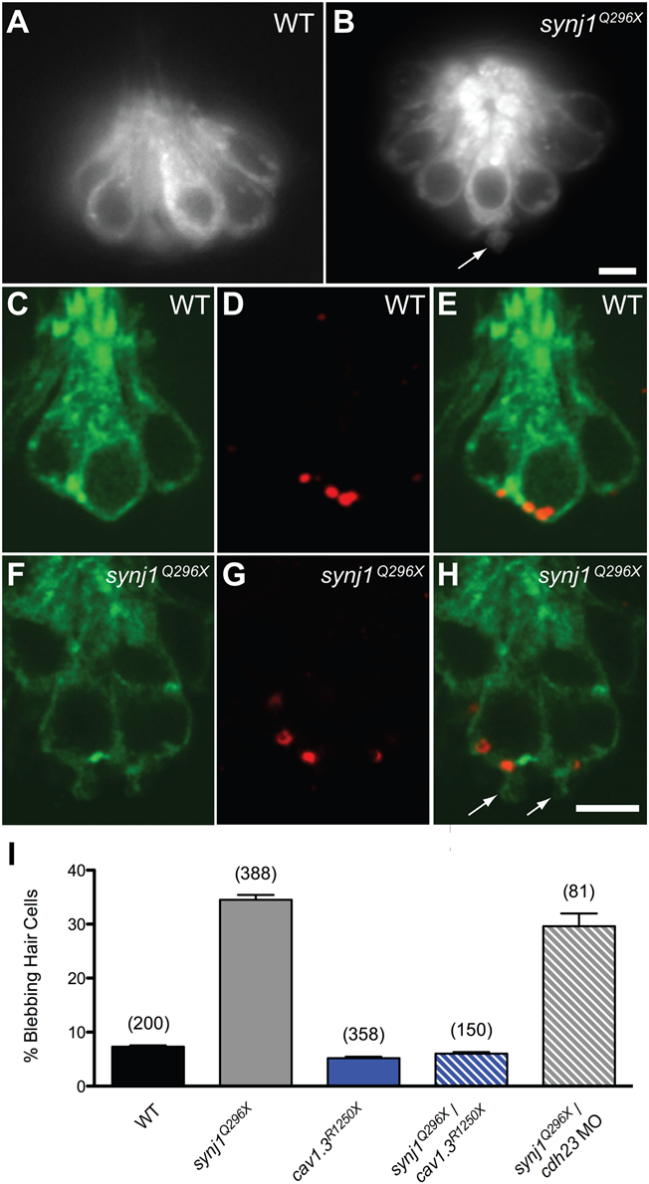
Basal blebbing in *synj1^Q296X^* hair cells occurs near synaptic ribbons and is Ca^2+^-dependent. FM 1–43 labeling of wild-type (A) and *synj1^Q296X^* (B) neuromast hair cells. Note the bleb or membrane protrusion at the base of a mutant cell (arrow). Single optical sections of membrane-targeted EGFP (*Tg(brn3c∶mgfp*)) expressed in wild-type (C) and mutant (F) hair cells. D, G) Ribeye-b immunolabel (red staining) of the same neuromasts, indicating ribbons. E, H) Overlays of membrane-targeted EGFP and Ribeye-b label. Arrows in panel H indicate blebs near the ribbon in mutant hair cells. I) Basal blebbing was Ca^2+^-dependent. Blebbing occurred minimally in neuromast hair-cells of wild-type larvae (black bar). In contrast, blebbing was significantly increased in *synj1^Q296X^* mutant hair cells (grey bar). Blebbing was minimal in *cav1.3^R1250X^* mutant hair cells (blue bar), as well as *cav1.3^R1250X^/synj1^Q296X^* double mutant hair cells (blue stripe bar). Blebbing still occurred in *synj1^Q296X^* mutants that were transduction-deficient by morpholino-mediated *cadherin23* knockdown (grey stripe bar). Numbers in parentheses above bars indicate number of hair cells examined. Scale bars, 3 µm.

Unlike mutant *synj1* photoreceptor ribbons, the ribbons in *synj1^Q296X^* hair cells are localized to the plasma membrane (Figure S1 and Figure 3H). However, we detected the presence of large basal membrane protrusions or blebs in approximately a third of mutant hair cells (34.5±0.9%, n = 388 cells; Figure 3). Large blebs emanating from mutant hair cells could be visualized in live, intact fish using the vital dye, FM1-43 (Figure 3B, arrow). Blebbing was also observed in inner ear or neuromast mutant hair cells transiently expressing soluble GFP (plasmid *myo6b∶GFP*; data not shown), and in mutant hair cells stably expressing a membrane-targeted form of GFP (*Tg(brn3c∶mGFP*); Figure 3F). When counterstained with anti-Ribeye b antibody, we observed that the extrusions of membrane occurred mainly near ribbons (Figure 3H). In addition, we examined the synaptic vesicle marker Vglut3 using immunofluorescence and saw that Vglut3 was present but not noticeably enriched in blebbing membranes (Figure S2). We also transiently expressed a GFP-tagged pleckstrin homology domain of phospholipase C_δ1_ protein that binds to PI(4,5)P2 [21] and did not note any obvious elevation in PI(4,5)P2 in mutant hair cells (data not shown).

To determine if the basal blebbing was dependent on synaptic transmission, we generated double mutant larvae that were homozygous for *synj1^Q296X^* and *cav1.3^R1250X^*. Cav1.3 L-type calcium channels mediate influx of calcium near hair-cell ribbons, causing the fusion of SVs [22],[23]. In *cav1.3a^R1250X^* single mutants, the amount of blebbing was comparable to wild-type levels (Figure 3I; *cav1.3^R1250X^*: 5.2±0.2%, n = 358 cells, 15 larvae; wild-type: 7.3±0.2%, n = 200 cells, 8 larvae). In contrast to *synj1^Q296X^* single mutants, we observed low levels of blebbing in *cav1.3^R1250X^/synj1^Q296X^* double mutants (Figure 3I; 6.0±0.3%, n = 150 cells, 6 larvae). Thus, the presence of Cav1.3a calcium channels was required for the blebbing phenotype observed in mutant *synj1* hair cells. In contrast to *cav1.3^R1250X^/synj1^Q296X^* double mutants, blebbing occurred independently of the presence of Cadherin 23, which is required for mechanotransduction (29.6±2.4%, n = 81 cells, 4 larvae; [24],[25]; Figure 3I). These results suggested that an imbalance of exo- and endocytosis led to blebbing in *synj1* mutant hair cells.

### Mutant *synj1* Hair Cells Produce Fewer Postsynaptic Spikes during High-Frequency Stimulation

We investigated the impact of *synj1* mutations on hair-cell transmission by stimulating neuromast hair cells and recording the evoked postsynaptic spikes from posterior lateral line ganglion (PLLg) cells [26]. Initially, we quantified the spontaneous activity of neuromast hair cells by recording action currents in the absence of water-jet stimulation. Wild-type afferent neurons displayed robust spontaneous activity (6.5±1.0 spikes/sec, n = 7). There was no significant difference in spontaneous activity in *synj1^Q296X^* neurons (7.0±0.9 spikes/sec, n = 7).

**Figure 4:**
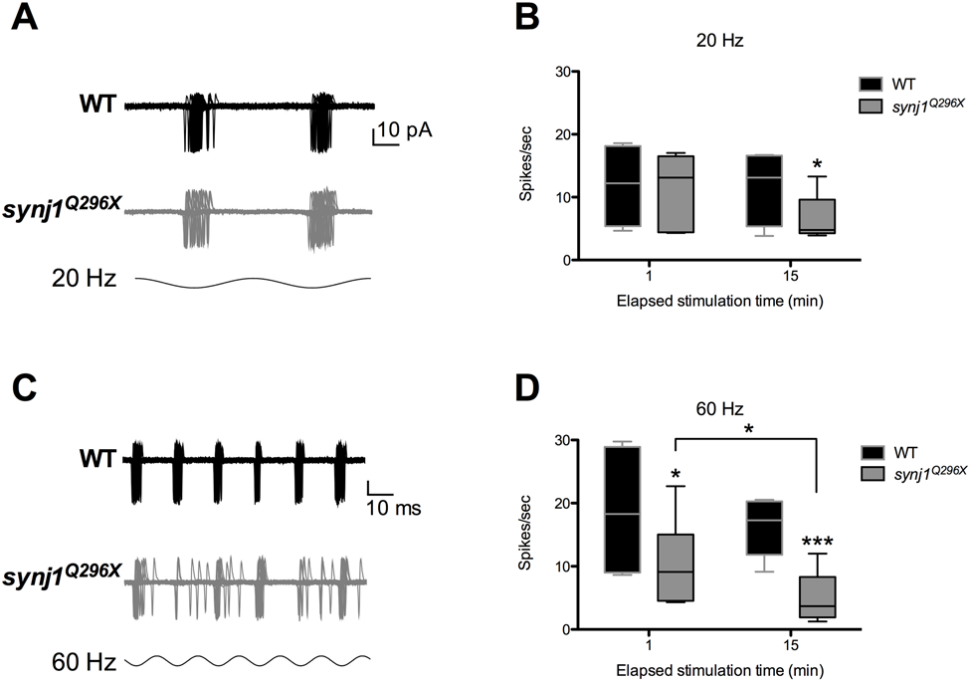
High frequency stimulation resulted in time-dependent reduction of the number of spikes in *synj1^Q296X^* afferent neurons. A) Spikes recorded from a wild-type (black traces) and *synj1^Q296X^* (grey traces) larva in response to 20 Hz stimulation. Shown is an overlay of 60 consecutive sweeps from an individual larva. B\) Box plot summarizing the spike rate (spikes per second) for 60 seconds of 20 Hz stimulation (black bars, wild-type; grey bars, *synj1^Q296X^*; includes larvae in A. C) Spikes recorded from a wild-type (black traces) and *synj1^Q296X^* (grey traces) larva in response to 60 Hz hair-cell stimulation. Shown are 60 consecutive sweeps overlaid from a single larva. D) Box plot summarizing the reduction in spike rate of *synj1^Q296X^* (grey bars) during 60 seconds of 60 Hz stimulation compared to wild-type neurons (black bars). Following 15 minutes of sustained stimulation the *synj1^Q296X^* mutant spike rate was reduced compared to both the initial rate and the wild-type rate.

To test whether mutant synapses displayed altered behavior following direct hair-cell activation, we mechanically stimulated neuromast hair cells at 20 Hz. We chose this rate or higher (see below) because lateral line hair cells are sensitive to frequencies of 1–150 Hz [27]. Stimulation at 20 Hz resulted in no significant difference in spike rate in the first 60 seconds of stimulation (mutant: 11.4±2.4 spikes/sec, n = 6; wild-type: 11.9±3.4 spikes/sec, n = 4; Figure 4A,B). Based on the previously described role of *synj1* in neurons, we hypothesized that sustained stimulation would fatigue the hair cell synapse. Indeed, stimulation of *synj1^Q296X^* hair cells at 20 Hz for 15 minutes resulted in a significant decrease in the number of spikes in *synj1^Q296X^* larvae compared to wild-type larvae (mutant: 6.6±1.5 spikes/sec, n = 6; wild-type: 11.7±3.0 spikes/sec, n = 4; Figure 4B)

To determine whether the reduction in activity seen in *synj1^Q296X^* afferent neurons was exacerbated at higher frequency, we increased the stimulation frequency to 60 Hz. Within the first minute of 60 Hz stimulation, mutant spike rate was largely reduced compared to wild-type (mutant: 10.3±2.3 spikes/sec, n = 8; wild-type: 19.4±3.2 spikes/sec, n = 7; Figure 4C,D). Next, we attempted to fatigue the synapse with 15 minutes of sustained 60 Hz stimulation. Following 15 minutes of continuous stimulation, the mutant spike rate (5.1±1.4 spikes/sec, n = 8) was significantly reduced compared to both the wild-type rate (15.7±1.7 spikes/sec, n = 7) and its own initial release rate (Figure 4D). To rule out the possibility that the sustained stimulation affected mechanotransduction, we obtained microphonic potentials before and after 15 minutes of 60-Hz stimulation. We saw no differences between wild-type and mutants in the recordings pre-and post-stimulation (Figure S3).

### SV Recycling Is Perturbed in Mutant *synj1* Hair Cells

**Figure 5:**
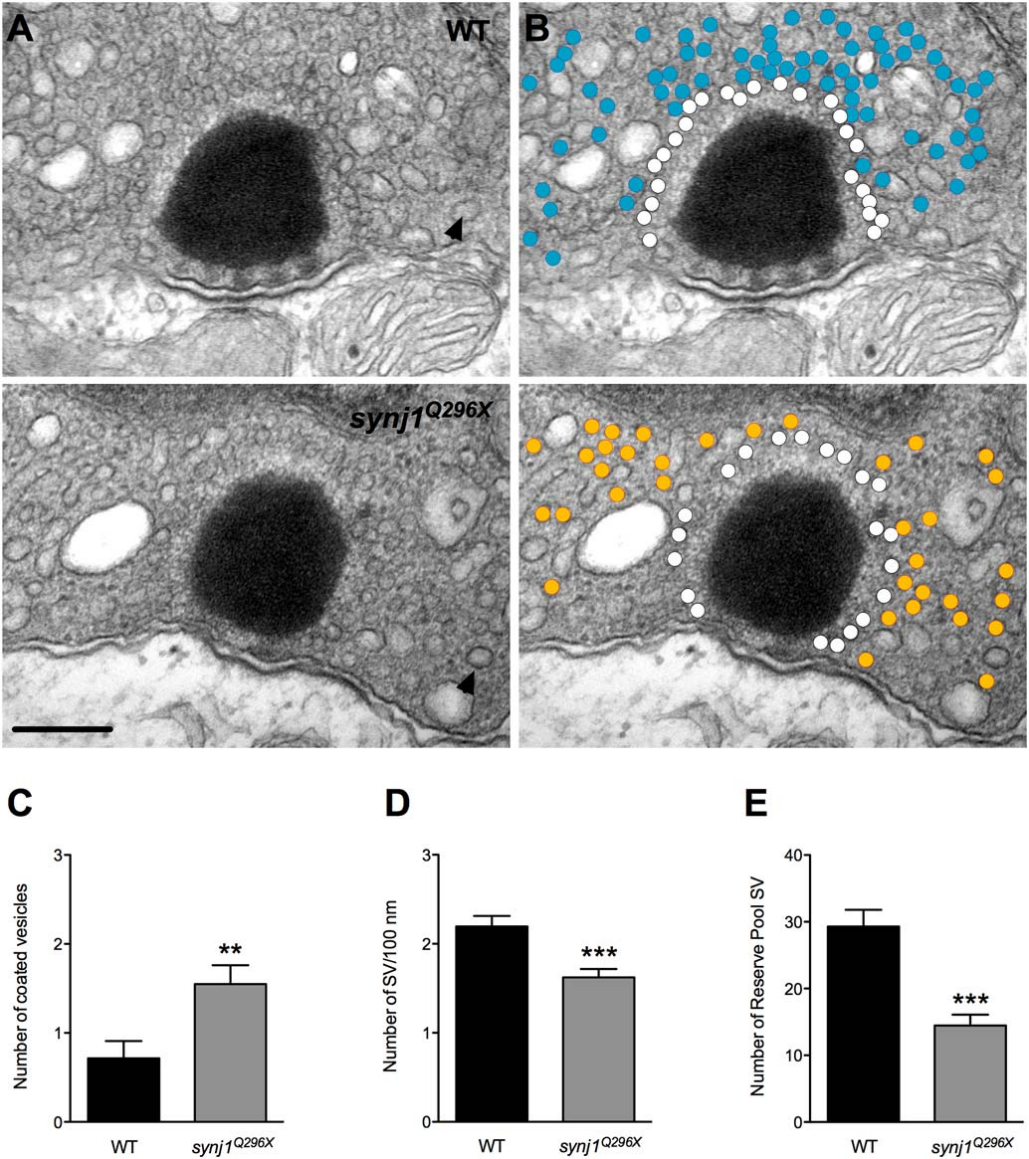
Impaired vesicle recycling at ribbon synapses in mutant *synj1* hair cells. A) TEM micrographs of neuromast ribbon synapses (upper panel: wild-type ribbon; lower panel: *synj1^Q296X^* mutant ribbon). B) Same micrographs as in (A) with white circles denoting tethered vesicles. Blue and orange circles highlight the reserve vesicles in the wild type and mutant synapses, respectively. Note the reduction of reserve pool vesicles in the mutant synapse. Arrow heads indicate examples of large coated vesicles. Scale bar, 200 nm. Number of large coated vesicles (C), tethered vesicles per 100 nm of ribbon perimeter (D), and reserve pool vesicles (E) within 450 nm of ribbon centers in neuromast hair cells (black bars: wild type; grey bars: *synj1^Q296X^*).

Given the previously described role for Synj1 in vesicle recycling and the decline of spiking in *synj1* mutants, we examined the various membrane components in stimulated wild-type and mutant hair cells using transmission electron microscopy. For these experiments, we stimulated whole larvae for 15 minutes at 60 Hz before fixation (see Methods). To quantify changes, we used single thin sections that featured the center of ribbon and counted structures within a 450 nm radius of the ribbon in neuromast hair cells (Figure 5). In mutant hair cells, we observed an increase in the number of large coated vesicles approximately 70 nm in diameter, which is larger than a typical SV with a diameter of 40 nm (*synj1^Q296X^* :1.6±0.2 large vesicles, n = 20 ribbons; wild-type: 0.7±0.2 large vesicles, n = 14 ribbons; Figure 5C). Large coated vesicles were also reported by Lenzi et al. upon maximal stimulation of frog hair cells (2002). Small clathrin-coated vesicles (CCVs; ≥50 nm) with a diameter similar to SVs were infrequently seen near the ribbon.

Previous *synj1* mutant studies in the worm and fly neuromuscular junction have suggested that the rundown of synaptic events in these mutants is the result of SV depletion [14],[28]. We counted the SV pools surrounding hair-cell ribbons on single sections as above. We define the first shell of SVs that decorate the ribbons as ribbon-tethered. With respect to this releasable pool of SVs, we observed a reduction of approximately 30% of ribbon-tethered SVs in mutant neuromast hair cells (mutant: 1.6±0.1/100 nm of ribbon perimeter, n = 30 ribbons; wild-type: 2.2±0.1/100 nm of ribbon perimeter, n = 18 ribbons; Figure 5D). We also observed far fewer reserve pool or non-tethered vesicles within 450 nm of ribbons in mutant neuromast hair cells (mutant: 14.5±1.6, n = 13 ribbons; wild-type: 29.3±2.5, n = 11 ribbons; Figure 5E).

With the exception of a two-fold increase of large coated vesicles, we noted only minor differences in membrane compartments of inner ear hair cells (data not shown). The relatively weak effect on inner ear hair cells is likely due to the 60 Hz stimulus being suboptimal for the inner ear maculae. In zebrafish larvae, an acoustic startle reflex can be elicited with frequencies ranging from 100–1200 Hz or potentially higher, with a higher sensitivity at ≥300 Hz [29]. The difference in sensitivity thus predicts the differential effect of the 60 Hz stimulus on inner ear and neuromast hair cells that we observed in our micrographs.

We also noted blebbing structures near ribbons in mutant hair cells in our micrographs. Three examples are shown in Figure S2. Protrusions or the appearance of destabilized membrane can be seen directly adjacent to the ribbon.

### Mutant *synj1* Hair Cell Output Is Delayed and Phase Locking Is Impaired

**Figure 6:**
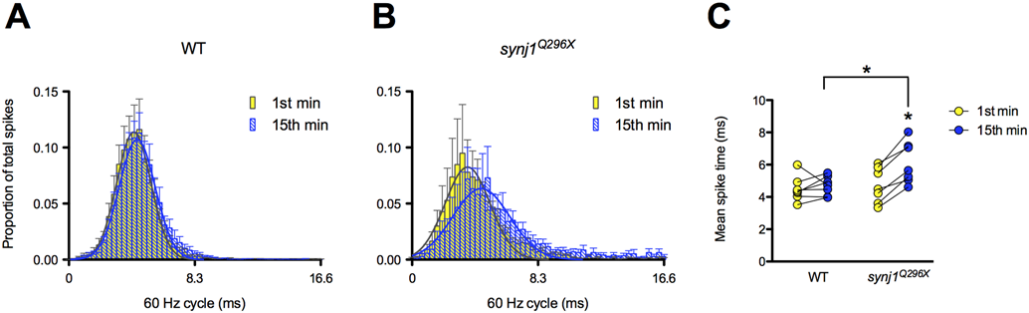
The mean timing of spikes in response to 60 Hz stimulation is delayed in *synj1^Q296X^* mutants. A, B) Histograms of 60 seconds of activity for (A) wild-type and (B) *synj1^Q296X^* afferent neurons in the first minute (yellow bars) and fifteenth minute (blue bars) of 60 Hz stimulation. The x-axis represents the length of one 60 Hz cycle in milliseconds. Curves were fit to Gaussian distributions (yellow and blue solid lines). C) Mean spike times of activity during 60 seconds of stimulation for individual wild-type and *synj1^Q296X^* mutants represented in (A). Lines connect the 1^st^ (yellow circles) and 15^th^ (blue circles) minute of stimulation for each larval recording. Following 15 minutes of sustained 60 Hz stimulation there was a significant delay in spike time compared to the first minute of stimulation in *synj1^Q296X^* as well as compared to wild-type after 15 minutes of stimulation.

In order to address whether the disruption in SV recycling affects the timing of hair-cell transmission to PLLg neurons, we examined spike timing in response to stimulation. First, for both wild-type and *synj1^Q296X^* larvae, we determined the timing of spikes relative to the start of each 60 Hz cycle for all spikes in the first and fifteenth minute of stimulation. The spike times from all larvae are represented in a histogram in Figure 6A–B. We then calculated the mean spike time for each PLLg recording (Fig 6C). In wild-type larvae, similar to the results for spike rate, there was no change in mean spike time from the first (4.5±0.3 ms, n = 7) to the fifteenth minute (4.7±0.2 ms, n = 7) of stimulation (Fig 6C). In contrast, the mean spike time was significantly delayed following 15 minutes of sustained stimulation of *synj1^Q296X^* hair cells (5.9±0.6 ms, n = 8) compared to the first minute (4.9±0.4 ms, n = 8; Figure 6C).

**Figure 7:**
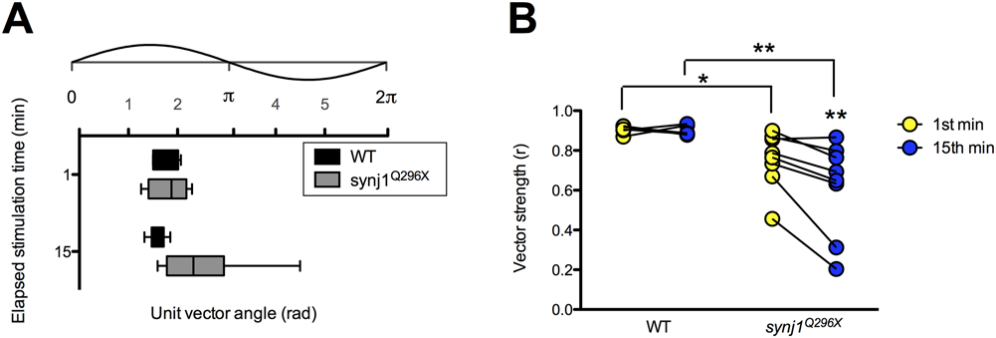
The fidelity of phase locking is decreased in *synj1^Q296X^* larvae. A) Box plot representing phase angles from the unit vectors described by all spikes in the 1^st^ and 15^th^ minute of 60 Hz stimulation from wild-type (black bars) and *synj1^Q296X^* (grey bars) larvae. Above the box plots is the corresponding 60 Hz stimulus cycle with phase scale in radians. B) Individual wild-type and *synj1^Q296X^* mutant vector strength (*r*) values for the 60 seconds of activity described in (A). Lines connect the 1^st^ (yellow circles) and 15^th^ (blue circles) minute of stimulation for each larval recording. Mutant vector strength was not only reduced at both time points compared to wild-type, but also from the 1^st^ to the 15^th^ minute of stimulation.

In addition to the overall delay in *synj1* mutant hair-cell response to stimulation, inspection of both the traces in Figure 4C and the histogram of mutant activity from Figure 6B revealed an impairment in the timing of release in response to the stimulus. Proper phase locking of hair-cell output to mechanical stimuli is a reflection of precisely timed transmission. In order to determine the fidelity of timing, we quantified the quality of phase locking of afferent activity by calculating its vector strength (see Methods). A vector strength (*r*) value of 0 implies essentially random activity, whereas a value of 1 describes perfect synchrony between stimulus and response. We determined the vector strength of 60 seconds worth of spikes in the first and fifteenth minute of stimulation in both wild-type and mutant afferent neurons. As expected, stimulus and response were tightly phase-locked in wild-type larvae at 60 Hz (1^st^ min: r = 0.88±0.03; 15^th^ min: r = 0.89±0.01; n = 7; Figure 7B). In contrast, *synj1^Q296X^* mutants, similar to the reduction in spike rate seen at 60 Hz, had significantly reduced vector strength in the first minute and a further reduction after fifteen minutes of sustained stimulation (1^st^ min: 0.75±0.05; 15^th^ min: 0.61±0.08; n = 8; Figure 7B). In addition, mutants had reduced vector strength at 20 Hz, but only after 15 minutes of stimulation (data not shown), further supporting the relationship between the reduction in spike rate and the impairment of spike timing (Figure 4B).

### Delay in Return of Spontaneous Activity Following Stimulation

**Figure 8:**
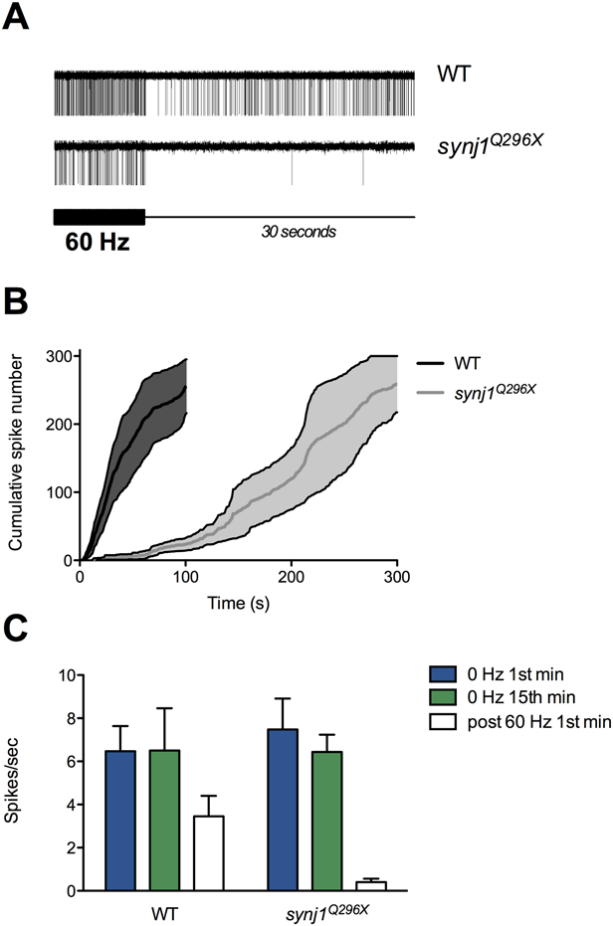
Return of spontaneous activity following 60 Hz stimulation is delayed in mutant *synj1* hair cells. A) Single continuous trace from wild-type (top) and *synj1^Q296X^* (bottom) showing the last 10 seconds of a 15-minute 60 Hz continuous stimulus (thicker portion of black scale bar) followed by the first 30 seconds of recording in the absence of stimulation (thin portion of black scale bar). Note the greater number of wild-type spikes during the 60 Hz stimulation portion of the trace. For the wild-type trace, the initial 30 seconds post-stimulation contains 200 spikes, while there are just two spikes in the mutant trace. B) Average cumulative frequency histogram of the first 300 spontaneous spikes recorded post stimulation in wild type (black line, dark shading represents error) and *synj1^Q296X^* (grey line, light shading represents error). C) Bar graph of spontaneous spike rate for wild type and *synj1^Q296X^* larvae for the 1^st^ (blue bars) and the 15^th^ (green bars) minute at 0 Hz and for the 1^st^ minute following 15 minutes of stimulation at 60 Hz (white bars). Note that the wild-type spontaneous spike rate approached that of unstimulated larvae while the mutant rate was near zero.

We define spontaneous activity as random spikes seen in the absence of mechanical stimulation of hair cells. Typically, after a period of direct stimulation, hair cells resume spontaneous output within seconds. We noted in our recordings after ceasing prolonged stimulation that mutants displayed a delay in return of spontaneous activity. To illustrate the phenomenon, we stimulated wild-type and mutant larvae at 60 Hz for 15 minutes, removed the stimulus and continued recording until greater than 300 spontaneous spikes were collected (Figure 8A). Wild-type larvae resumed spontaneous activity within 5±2 seconds (average of first spike times; n = 4; Figure 8A). In contrast, the average time for recovery of first spikes in *synj1^Q296X^* mutants was six-fold slower (31±17 seconds, n = 4; Figure 8A). Comparison of the time elapsed for the first 300 spontaneous spikes to occur in both wild-type and mutant larvae gave an approximate rate of recovery (Figure 8B). Mutants took roughly three-fold longer to generate 300 spikes, with the majority of output delayed greater than two minutes post-stimulation. To further describe the delay in recovery of spontaneous activity, we determined the spike rate in the first 60 seconds post-stimulation in both wild-type and *synj1^Q296X^* larvae (Figure 8C). While the wild-type post-stimulation rate had nearly resumed normal levels, the firing rate in *synj1^Q296X^* mutants remained near zero (mutant: 0.4±0.2 spikes/sec, n = 4; wild-type: 4.0±1.3 spikes/sec. n = 4).

## Discussion

In this article, we report three new *synaptojanin1* mutant alleles in zebrafish: *synj1^Q296X^*, *synj1^W943X^*, and *synj1* ^c.188+2T>A^. The first two alleles generate truncated products; the third allele is a splice-site mutation that eliminates the translation start site. We found that the zebrafish *synj1* gene is highly expressed in the nervous system and retina as previously described by Van Epps [19], but we also observed expression in hair cells of the larval inner ear and lateral line organ. Synj1 has been implicated in both early and later steps in classical clathrin-mediated endocytosis (CME) [30], and one might expect that CME would occur near ribbons where the greater part of exocytosis takes place [31]. Indeed, CME was observed in goldfish retinal bipolar neurons [32],[33], but molecular analysis of this type of membrane retrieval has not been examined in hair cells. Alternatively, there is evidence that bulk endocytosis occurs within the vicinity of the hair-cell ribbon. Following sustained depolarizations, large endocytic profiles and cisternae, which are hallmarks of bulk endocytosis, have been observed near hair-cell ribbons [12]. This type of membrane retrieval is especially apparent during prolonged stimulation of hair cells. We find that Synj1 function was most critical during high demands on synaptic transmission as the *synj1* mutant phenotype was more severe during sustained stimulation at higher frequencies (see below). At mutant ribbon synapses there was an increase in large coated vesicles and decreased numbers of reserve pool vesicles. Apparently, the disrupted step in membrane recycling in *synj1* mutant hair cells was the breakdown of endocytic structures into small synaptic vesicles. Therefore, it remains a possibility that Synj1 is involved in one or several steps of bulk endocytosis.

Lesions in *synj1* also cause similar defects in central or NMJ synapses of other species. *Caenorhabditis elegans (C. elegans)*, *Drosophila melanogaster (Drosophila)*, and mouse Synj1 mutants have been reported to show slowed endocytosis [14],[34],[35], depletion of SVs, accumulation of clathrin-coated vesicles (CCVs), and aggregation of cortical actin [16],[28]. A unique feature of the mutant zebrafish phenotype was the presence of basal blebs in hair cells, suggestive of an imbalance of exo- and endocytosis. Indeed, the dependence of the hair-cell phenotype on the presence of Cav1.3 channels suggests that exocytosis leads to blebbing near ribbons. Another unique feature was the increase in large coated vesicles, as opposed to small coated vesicles. However, an increase of endosome-like structures is also noted in *C. elegans* unc-26 synapses [28]. These authors suggest that UNC-26/SYNJ may be involved in converting endosomal compartments into synaptic vesicles. Moreover, a subpopulation of large vesicles is observed among the remaining SVs in *Drosophila synj1* mutant NMJ terminals, suggesting a defect in vesicle formation [14]. Collectively, these phenotypes support the notion that Synj1 participates in multiple steps of membrane recycling.

The apparent defect in membrane recycling in mutant *synj1* hair cells had the highest impact on the reserve pool—the number of vesicles was reduced by 50%. A smaller reserve pool is consistent with an impairment of bulk retrieval [36]. We speculate that the reduction of the reserve pool at neuromast ribbons in turn resulted in fewer tethered SVs. For our TEM experiments, we used a 60 Hz whole-larval stimulation to simulate the conditions of our physiological recordings. Whether this form of stimulation replicates the direct sinusoidal waterjet stimulation remains to be determined. Nevertheless, this rate of stimulation falls into the physiological range of lateral line hair cells [27]. Our experiments measuring the onset of spontaneous activity after prolonged stimulation suggest that spontaneous transmission in *synj1^Q296X^* mutants is fatigable and requires several minutes to fully recover. This lengthy delay in the return of spontaneous release may reflect a preference to restock the reserve pool before un-evoked SV release is allowed to resume.

Our recordings of evoked action currents in post-synaptic neurons revealed that the mutant hair-cell synaptic properties were relatively normal during short periods of low frequency (20 Hz) stimulation. The requirement for Synj1 became apparent only when high demands on transmission were made. A decrease in the rate of transmission emerged after tens of minutes of stimulation at 20 Hz and occurred without delay at a three-fold higher frequency (60 Hz). Under these conditions, we saw a 50% or greater reduction in the number of spikes. Furthermore, at the higher frequency of 60 Hz, we observed two additional phenotypes - a delay in the spike timing and an impairment of phase locking to the stimulus. Following prolonged periods of stimulation, wild-type hair cells displayed both tight phase locking and no change in the average spike response time to stimulation. In contrast, a clear delay of spiking occurred in mutant synapses with some spikes delayed by as much as 8 ms within a 60 Hz cycle. When quantified for the degree of synchrony between the response and stimulus, mutants also displayed a significant reduction in the quality of phase locking.

The defect in phase locking, combined with the decrease in tethered vesicles and reserve pool vesicles, suggests that refilling of the ribbon or formation of SVs from endocytic compartments was rate limiting in mutant hair cells. Our results support the idea that vesicle numbers are critical for temporal fidelity of SV release at ribbon synapses [11]. Other possibilities may also explain the loss of phase locking, such as an indirect effect on the exocytic machinery. For example, prolonged stimulation could lead to diffusion or mislocalization of active zone proteins such as Cav1.3a channels, which mediate the calcium influx required for SV fusion. In our hands, labeling of hair cells stimulated for 15 minutes at 60 Hz with antibodies against Cav1.3a and Ribeye did not reveal any differences between wild type and mutant hair-cells (Sheets, L. and Nicolson, T., unpublished observations). Another possibility is a more subtle disruption of the attachment of the ribbon to the plasma membrane. While a slight displacement of the ribbon would likely delay vesicle fusion, it isn't clear how it would disrupt phase-locking fidelity. Furthermore, ribbon mislocalization was not apparent in our immunofluorescence images or TEM micrographs. Another possibility is that the third or so hair cells with basal blebs near ribbons may account for the loss of phase locking. Perhaps the destabilization of the plasma membrane in these cells leads to abnormal fusion of SVs. A further possibility is the presence of an endocytic defect on the post-synaptic side of mutant ribbon synapses. A recent study describes the failure to endocytose pHluorin-tagged α-amino-3-hydroxy-5-methylisoxazole-4-proprionic acid (AMPA) receptors in cultured *synaptojanin*-KO hippocampal neurons [37]. In this study, an increase of miniature excitatory postsynaptic currents (mEPSC) is attributed to an increase in surface exposure of AMPA receptors. An increase in exposed AMPA receptors would likely lead to an increase in spike rates. A recycling defect of AMPA receptors in *synj1^Q296X^* PLLg neurons is possible, however, rather than observing increased spontaneous or stimulus-evoked spiking, we saw a decrease in post-synaptic activity.

In actively swimming larvae, *synj1* mutants suffer from an easily observable balance defect. If swirled continuously in a stream of water, the severity of the vestibular defect increases, suggesting fatigue of synaptic transmission. Such an effect on behavior is consistent with our recordings of afferent neuron activity where a decrease in synaptic ribbon function was seen during sustained stimulation. Similarly, the reduction of a robust response in the VOR test presumably resulted from fewer synaptic transmission events during stimulation of vestibular hair cells. Light tapping on a Petri dish of *synj1* mutants will elicit a response, which suggests that their acoustic startle reflex is intact (data not shown). Perhaps continuous stimulation of the auditory system in *synj1* mutants would also affect this acoustic startle reflex. Whether hearing is impaired in terms of sensitivity or frequency range in mutant *synj1* zebrafish awaits further study.

Our results suggest that maintenance of SV pools is critical for reliable and temporally precise transmission at the hair-cell ribbon synapse. Transduction in hair cells induces the release of thousands of vesicles during strong stimulation, which poses a particular challenge to endocytic retrieval mechanisms. Based on our data, we propose that Synj1 plays a critical role in facilitating vesicle recycling and if impaired, vesicle recycling not only affects the number of vesicles released at ribbon synapses, but also the timing of release.

## Materials and Methods

### Fish Strains

Mutant alleles were maintained in Tübingen or Top Long Fin wild-type backgrounds. The transgenic lines *Tg(brn3c∶mGFP)* and *Tg(neurod∶GFP)* were previously described [26],[38]. In addition, the *gemini/cav1.3^R1250X^* (*tc123d* allele) mutant was previously described [22]. The *cdh23*-MO (targeted to the ATG start site) was also previously described [25]. For all experiments, the genotype of larvae was confirmed.

### VOR Experiments and Analyses

Larvae were tested as previously described with some modifications [26]. Zebrafish larvae placed in 2% low-melting agarose dorsal side up on a cover glass were mounted facedown on a vertical sample platform. The platform was driven by a motor (BE2310J-NPSN, Parker Hann. Inc.), controlled by a servo controller (GV6K-U3E, Parker Hann. Inc.), at a frequency of 30°/s and amplitude of ±60°. Movement was controlled by Motion Planner software (Parker Hann. Inc). Eye movements were recorded by digital camera (DCM130; Hangzhou Scopetek Opto-Electric, Zhejiang, China) through a 10× objective lens (Mitutoyo, Neuss, Germany), illuminated by infrared (800 nm), at 7–9 frames per second and at a resolution of 1024×768 dpi (ScopePhoto). In order to read platform position directly from video, a servo controller generated 100 ms pulse, timed 100 ms after the motor reached an edge, briefly shut down the illumination. Video was processed in MATLAB (Mathworks, Natick, MA), using functions in the Image Processing Toolbox. The ratio (R) of the long and short axes of the fish eye was extracted from each frame. For quantifying eye movement, the relative ratio change was calculated as (R – mean(R))/mean(R). Since the eye movement represented a response to sinusoidal stimulation, the magnitude of eye movement was the power at the stimulation frequency (determined by discrete Fourier transformation).

### Molecular Biology

Genomic DNA and mRNA were extracted from day 5 zebrafish larvae by standard methods. First-strand cDNA was generated with the SuperScript III First-Strand Synthesis System (Invitrogen) and an oligo d(T) primer, and the alleles are designated as follows: *synj1*c188+2T>A (formerly called IG459), donor splice site, exon2; *synj1^Q296X^* (formerly called JV039), post sac1-domain truncation; *synj1^W943X^* (formerly called *synj1^HT039^*) post IPPc domain truncation. Primers used for sequencing were previously described [19].

### Neuromast RT-PCR

Neuromast hair-cell cores were obtained using the same glass pipet used for hair-cell stimulation filled with 5 µl of lysis buffer. The pipet was attached via tubing to a hand-held 60 mL syringe. Upon positioning the pipet against a rosette of hair cells, gentle suction was applied while at the same time raising the pipet away from the larva. The entire hair-cell group was usually collected within seconds. On average 5 neuromasts (∼50 hair cells) were pooled, transferred to a PCR tube and then cDNA extraction and amplification was performed.

### In Situ Hybridization

Digoxygenin-labeled riboprobes were generated with the DIG Labeling Kit (Roche) from *synj1* (nt 1–692) cDNA that had been cloned into pCR-II (Invitrogen). In situ hybridization was conducted as previously described with 200 ng of probe [25]. Larvae at 72 hours post fertilization were imaged on a Zeiss Axio bright-field microscope using a 10× or 20× dry lens objective. Images were acquired via an AxioCam MRc5 color digital camera using Axiovision software and exported as TIFF files to Adobe Photoshop for analysis.

### Immunofluorescence

Hair cells were labeled with either anti-Ribeye b or anti-Vglut3 antibodies as previously described [26].

### Electron Microscopy and Vesicle/Endosome Counting

Mutant *synj1* and wild-type larvae were stimulated on a mini-shaker (Bruel and Kjaer) at 0.5 V at 60 Hz for 15 minutes [29]. Larvae were fixed immediately in 3% glutaraldehyde and 1.5% paraformaldehyde in 0.1 M phosphate buffer for ≥12 h, stained with 1% osmium, dehydrated in ethanol and embedded in araldite. Transverse thin sections (60–80 nm) through the ear and neuromasts were imaged on a Phillips CM100 Electron Microscope. Images of ear and neuromast hair-cell ribbons were collected at 25,000×–64,000× magnification. Images were acquired on an analog camera and negatives were scanned into Adobe Photoshop at 1200 dpi. Sections of stimulated larvae were collected from a total of 5 wild-type and 5 mutant specimens. We generated ≥5 sections per specimen, and each section contained ≥3 hair cells. Our analysis was confined to those ribbons that were directly adjacent to a plasma membrane connected to an afferent bouton. Tethered synaptic vesicles (30–50 nm diameter) were counted as described previously [26]. Same-sized vesicles that were not tethered were counted as reserve pool vesicles. To examine the same cross-sectional area in each section (cut through the center of ribbons; off-center sections were not counted), a circle with a radius of 450 nm was created using ImageJ (NIH) and centered on the ribbon body in images at the same magnification (64,000×). We chose 450 nm because longer distances often included large mitochondria or nuclei within the cross-sectional area.

### Electrophysiology and Postsynaptic Recordings

The recording setup was similar to that described previously [26]. Briefly, day 5 larvae were anesthetized, mounted and microinjected in the heart with 125 µM α-bungarotoxin to suppress muscle activity. Larvae were then rinsed and maintained in normal extracellular solution (in mM: 120 NaCl, 2 KCl, 2 CaCl2, 1 MgCl2 and 10 HEPES, pH 7.3). The recording micropipette was also filled with normal extracellular solution. Pipettes were fabricated (P-97, Sutter Instruments) from borosilicate glass with tip diameters of approximately 1 µm and resistances between 15 to 20 MΩ. Signals were collected with an Axopatch 200B, a Digidata 1440A and pClamp 10 software (Molecular Devices). Extracellular currents were collected in voltage clamp mode, filtered at 1 kHz and sampled at 100 µs/pt. The recording electrode and waterjet pipet were positioned with stepper-motor micromanipulators (Sutter Instruments). The waterjet pipet was positioned ∼100 µm from a given neuromast and displacement of the cupula was verified by eye. The recording electrode was positioned within the posterior lateral line ganglion against a cell body of choice. Following observation of spontaneous spiking, the corresponding neuromast was located by detection of phase-locked spiking upon waterjet stimulation. The microphonic recordings were obtained as in [26].

### Mechanical Stimulation

Neuromast hair cell stimulation was performed as described previously [24]. In short, we stimulated hair cells with a pressure clamp (HSPC-1, ALA Scientific, New York) attached to a glass micropipette (tip diameter ∼30 µM) filled with normal extracellular solution. In order to elicit activation of hair-cell transduction channels, the waterjet was oriented along the long body axis in order to deflect the cupula laterally. The pressure clamp was driven by a sinusoidal voltage command, which resulted in bidirectional deflection of the cupula. For a given experiment, the voltage command was driven concurrently by pClamp via a second analog output from the Digidata. During each experiment the quality and timing of the waterjet pressure was monitored via a feedback sensor located on the HSPC-1 headstage. For each experiment, this feedback pressure signal was collected in Clampex alongside the extracellular current recording and used for alignment of the apparent stimulus phase to recorded postsynaptic currents. Stimulus trains of 0, 20 and 60 Hz were delivered in 1-second episodes with minimum time between episodes.

### Signal Analysis

Data were analyzed and plotted with Axograph X (Axograph Scientific) and Prism 5 (Graphpad) software. The number of spikes per second was averaged from the total number of spikes in 60 consecutive episodes. Individual spike time was calculated to correspond with each stimulus sine-wave cycle (i.e. t = 0 at the start of each sine-wave period). This resulted in every spike time occurring within 0 and either 50 (20 Hz) or 16.66 (60 Hz) milliseconds. To quantify the synchronization of the hair cell response to stimulus phase, we converted the individual spike times to unit vectors with specific phase angles (see Figure 7A). The equation for vector strength (*r*) was then used as a measure of the degree of phase locking between stimulus and response [39]. Values in the text are expressed as mean±SEM. Statistical significance was determined by using either paired or unpaired Student's t tests, as appropriate, and are indicated in figures as * p<0.05, ** p<0.01, and *** p<0.001.

## Supporting Information

Figure S1 Synaptic ribbons in *synj1^Q296X^* hair cells are localized to basolateral regions adjacent to lateral line nerve fibers. Ribbons in wild-type (A) and *synj1^Q296X^* (B) neuromasts were labeled with anti-Ribeye b antibody (red staining). Nerve fibers were visualized using GFP fluorescence stably expressed by a neurod promoter (green staining). Shown are maximum projections of confocal sections from a top down view. Scale bar, 3 µm.
(1.65 MB TIF)

Figure S2 Examples of loss of integrity of the plasma membrane near mutant *synj1^Q296X^* ribbons. (A) Confocal optical sections of inner ear hair cells labeled with anti-Vglut3 antibody in wild type and *synj1^Q296X^* larvae. (B) Left panel, blebbing seen in an *synj1^Q296X^* inner ear hair cell at the TEM level. Right panels, two ribbons in *synj1^Q296X^* neuromast hair cells. Arrows indicate membrane protrusions or areas that appear to the disintegrating. Scale bar, 125 nm in left panel; 100 nm in top right panel; 200 nm in lower right panel.
(0.19 MB JPG)

Figure S3 Microphonic potentials are normal in *synj1^Q296X^* mutants. (A) Upper panel shows a representative wild-type microphonic recording in response to 20 Hz stimulation. The trace is an average of 200 consecutive sweeps. Lower panel shows a representative average trace from mutant following 15 minutes of sustained 60 Hz stimulation of the same neuromast. (B) The peak to peak magnitude of the microphonics were obtained from individual larvae identified in the parentheses above each condition. Mutants and mutants following stimulation were not significantly different than wild type.
(0.60 MB TIF)
