# Supplementary figures and images for: synaptojanin1 Is Required for Temporal Fidelity of Synaptic Transmission in Hair Cells
Source: PLoS Genet. 2009 May 8;5(5):e1000480. doi: 10.1371/journal.pgen.1000480 (PMC2673039; doi:10.1371/journal.pgen.1000480)

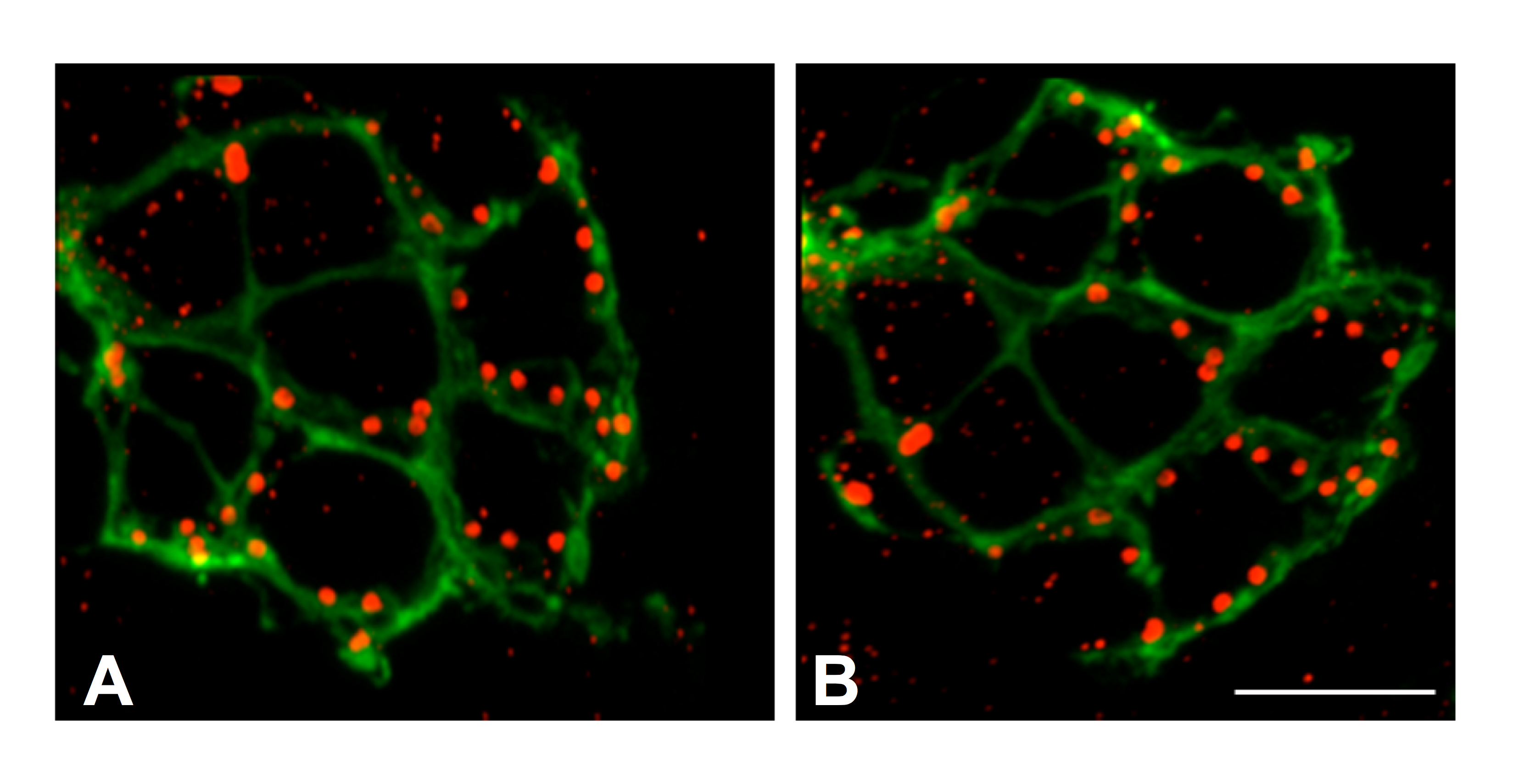

Supplement: Figure S1 — Synaptic ribbons in synj1Q296X hair cells are localized to basolateral regions adjacent to lateral line nerve fibers. Ribbons in wild-type (A) and synj1Q296X (B) neuromasts were labeled with anti-Ribeye b antibody (red staining). Nerve fibers were visualized using GFP fluorescence stably expressed by a neurod promoter (green staining). Shown are maximum projections of confocal sections from a top down view. Scale bar, 3 µm. (1.65 MB TIF) [file pgen.1000480.s001.tif]

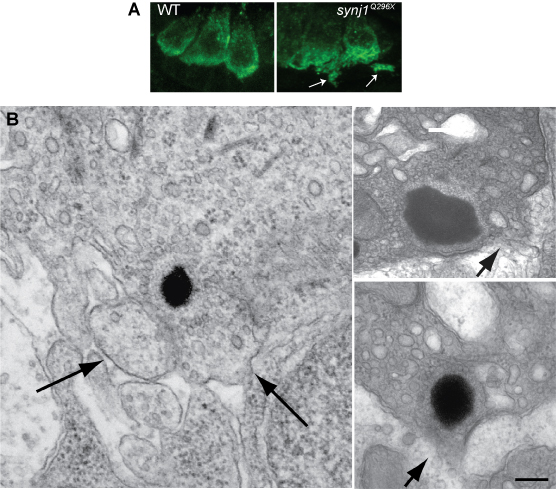

Supplement: Figure S2 — Examples of loss of integrity of the plasma membrane near mutant synj1Q296X ribbons. (A) Confocal optical sections of inner ear hair cells labeled with anti-Vglut3 antibody in wild type and synj1Q296X larvae. (B) Left panel, blebbing seen in an synj1Q296X inner ear hair cell at the TEM level. Right panels, two ribbons in synj1Q296X neuromast hair cells. Arrows indicate membrane protrusions or areas that appear to the disintegrating. Scale bar, 125 nm in left panel; 100 nm in top right panel; 200 nm in lower right panel. (0.19 MB JPG) [file pgen.1000480.s002.jpg]

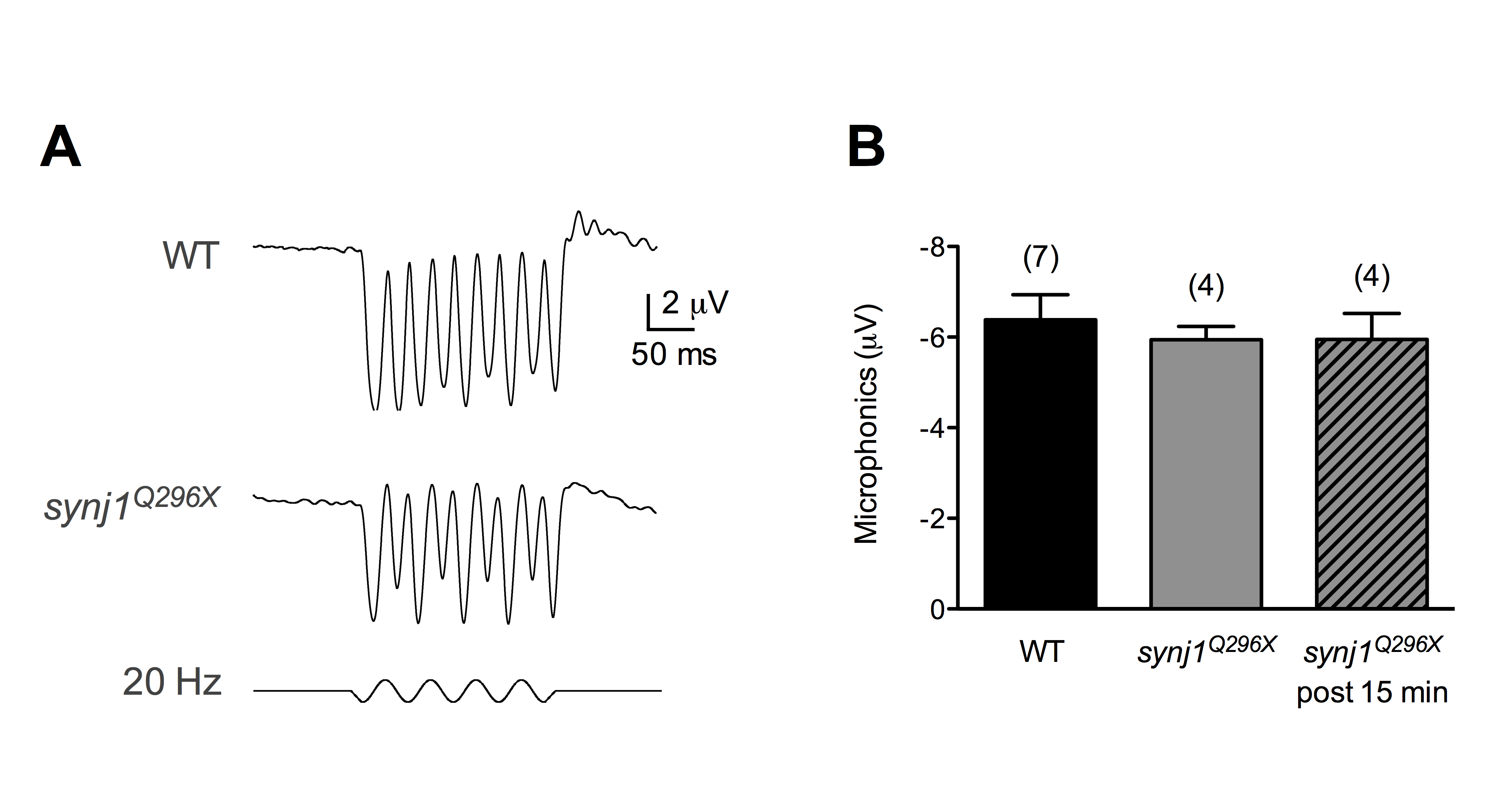

Supplement: Figure S3 — Microphonic potentials are normal in synj1Q296X mutants. (A) Upper panel shows a representative wild-type microphonic recording in response to 20 Hz stimulation. The trace is an average of 200 consecutive sweeps. Lower panel shows a representative average trace from mutant following 15 minutes of sustained 60 Hz stimulation of the same neuromast. (B) The peak to peak magnitude of the microphonics were obtained from individual larvae identified in the parentheses above each condition. Mutants and mutants following stimulation were not significantly different than wild type. (0.60 MB TIF) [file pgen.1000480.s003.tif]
